# Organized Plasmonic Clusters with High Coordination Number and Extraordinary Enhancement in Surface-Enhanced Raman Scattering (SERS)**

**DOI:** 10.1002/anie.201207019

**Published:** 2012-11-04

**Authors:** Nicolas Pazos-Perez, Claudia Simone Wagner, Jose M Romo-Herrera, Luis M Liz-Marzán, F Javier García de Abajo, Alexander Wittemann, Andreas Fery, Ramón A Alvarez-Puebla

**Affiliations:** Physikalische Chemie II, Universität Bayreuth, Universitätsstrasse 3095440 Bayreuth (Germany) E-mail: andreas.fery@uni-bayreuth.de; Colloid chemistry, University of Konstanz, Universitätsstrasse 1078464 Konstanz (Germany) E-mail: alexander.wittemann@uni-konstanz.de; Centro de Nanociencias y Nanotecnología, UNAM, km 107 Carretera Tijuana-EnsenadaC.P. 22860, Ensenada B.C. (México); Departamento de Química-Física, Universidade de Vigo36310 Vigo (Spain); Ikerbasque, Basque Foundation for Science, 48011 Bilbao (Spain) and Centre for Cooperative Research in Biomaterials (CIC biomaGUNE)Paseo de Miramón 182, 20009 San Sebastián (Spain); Instituto de Quimica-Fisica Rocasolano-CSICSerrano 119, 28006, Madrid (Spain); ICREA (Catalan Institution for Research and Advanced Studies), Passeig Lluís Companys 23, 08010 Barcelona (Spain) and Department of Electronic Engineering & Center for Chemical Technology of Catalonia, Universitat Rovira i VirgiliAvda. Països Catalans 26, 43007 Tarragona (Spain)

**Keywords:** enhancement factors, hotspots, nanoparticles, plasmons, surface-enhanced Raman scattering

Noble metal nanoparticles exhibit optical excitations known as surface plasmons that produce large enhancement of the local light intensity under external illumination, particularly when the nanoparticles are arranged in closely spaced configurations.^1^ The interparticle gap distance^2^ plays a critical role in the generation of hotspots with high electromagnetic fields, and thus such assembled nanoparticles find application to ultrasensitive detection, for example through surface-enhanced Raman scattering^3^ (SERS) and nonlinear optics, among other feats.^4^ Controlled assembly using colloidal chemistry is an emerging and promising field for high-yield production of metal nanoparticle clusters with small interparticle gaps.^5^ However, most of the reported methods rely on the use of nucleic acids or other organic molecules as linking elements,^6^ which yield long separation distances and thus weak plasmon coupling. Additionally, only simple clusters, such as dimers and trimers, have been efficiently synthesized. Herein, we report the controlled assembly of gold nanospheres into well-defined nanoparticle clusters with large coordination numbers (up to 7) and high symmetry. We further demonstrate ultrasensitive direct and indirect SERS sensing, thus corroborating the outstanding optical performance of these clusters with robust enhancement factors that are over three orders of magnitude higher than those of single particles.

The optical response of plasmonic particles upon light irradiation is mainly determined by particle size and shape, as well as the dielectric environment and geometrical arrangement.2b, 7 Recent advances in wet-chemical synthesis enable the fabrication of nanostructures with engineered complex size, shape, and composition.^8^ However, the colloidal synthesis of organized associations of these particles to fully exploit their plasmonic couplings remains a challenge, despite increasing efforts to produce monodisperse stable colloidal clusters. Pioneering work^9^, ^10^ demonstrated the possibility of obtaining nanoparticle dimers and trimers by using molecular linkers such as thiolated nucleic acids or other long organic molecules. This molecular linking approach is still frequently used, even though the final product is a mixture of clusters with different numbers of particles. Actually, numerous variations of this method exist for the production of homogeneous samples containing dimers and other geometries with low coordination numbers;6b, 11 with this approach a purification process is always required, typically including sedimentation,^12^ electrophoresis,^13^ and/or density gradient centrifugation.^14^ However, the application of these clusters to plasmonics in general, and to SERS in particular, is still limited because of three severe constraints. First, the use of thiolated linkers chemically passivates the plasmonic surface, thereby inhibiting the adsorption of other target species; second, the linker itself produces a SERS signal. These issues are crucial, considering that the thiolated molecule is placed precisely at the hotspot. And finally, perhaps the most important limitation is the size of the molecule: linkers based on nucleic acids require a minimum size of approximately 10 nm, leading to interparticle distances that are too long to produce effective hotspots. To avoid this drawback, a novel approach based on the linkage and purification of dimers and subsequent in situ overgrowth was recently reported.^15^ Despite the improvement, this method is still subject to vibrational contamination from the DNA linkers, and it has been only demonstrated for dimers.

Other approaches for the generation of dimers in colloidal solution include the use of asymmetric functionalization or polymer ligands. In the former, particles are functionalized with different capping agents, which can chemically bind to each other through simple reactions. The most popular choice is the functionalization of separate nanoparticle batches with amino and carboxylic groups. After mixing, carbodiimide chemistry serves to bind the two different fragments, thereby leading to formation of particle clusters.^16^ This approach, however, is affected by the same problems as those observed when using DNA or organic linkers. In contrast, polymer ligands exploit electrostatic interactions between a polyelectrolyte and the particles. This method has proven effective to form nanoparticle dimers with poly(vinyl pyrrolidone), poly(ethylene glycol), and poly(methyl methacrylate).^17^ The use of polymers instead of DNA or organic linkers not only allows efficiently gluing colloidal particles with smaller interparticle gaps,^18^ but also concentrating the analytes^19^ within close proximity to the plasmonic surface while avoiding vibrational signal contamination, because the polymers display low Raman cross-sections.^20^

We combine here these two properties by resorting to block copolymers based on poly(ethylene oxide)-b-poly(propylene oxide)-b-poly(ethylene oxide) (Pluronic F-68, PF68) to produce highly symmetric gold nanoparticle clusters with coordination numbers (CNs) up to 7. The resulting clusters are efficiently separated by density gradient centrifugation into homogeneous aliquots of dimers (CN=2), trimers (CN=3), tetramers (CN=4, mostly forming tetrahedra), and a complex mixture of trigonal bipyramids; octahedra; and pentagonal bipyramids (CN= 5, 6, and 7). The optical properties of the resulting materials are extensively characterized and theoretically modeled to explain their unique optical performance for electromagnetic field enhancement.

The assembly of the colloidal nanoparticles into clusters comprises initially the fabrication of monodisperse spherical gold nanoparticles (NPs) of average diameter of approximately 50 nm by a seed-mediated approach.^21^ After preparation, the NPs capped with cetyl trimethylammonium bromide (CTAB) show a zeta potential of +48 mV. The NPs are then concentrated and transferred into an aqueous solution of PF68, which leads to a reversal of the zeta potential to −19 mV. The colloidal dispersion of gold NPs coated with PF68 is then emulsified with toluene by using an ultrasonic homogenizer. This procedure yields narrowly dispersed emulsion droplets bearing nanoparticles at their surface.^22^ Packing of the NPs into clusters is induced by gradual toluene evaporation (in a rotary evaporator). The isotropy of the starting particles enables the construction of highly symmetric clusters. Finally, the separation of the different populations of clusters is achieved by density gradient centrifugation. Separation is then induced by the specific sedimentation velocity of each different cluster under centrifugation, giving rise to the typical stripes shown in Figure 1 a. The different fractions of clusters can then be collected using a piercing unit and separated again to ensure purity. All these processes lead to the complete separation of clusters with coordination numbers of 2, 3, and 4 (Figure 1 b). Owing to the close density values for higher CNs (5–7), gradient centrifugation does not have enough resolution and these clusters are obtained as a mixture within the same stripe.

**Figure 1:**
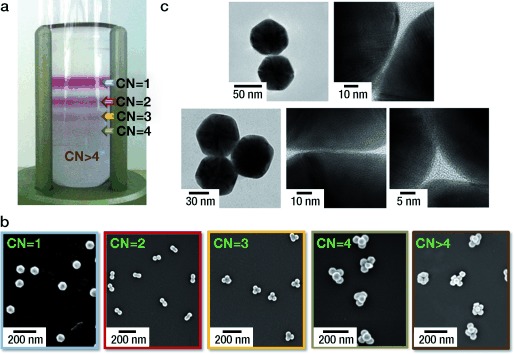
Preparation of gold nanoclusters with high coordination numbers. a) Density gradient separation to obtain a specific population from the initial mixture of clusters. b) SEM micrographs of the different cluster populations obtained after careful fractionation of the corresponding stripes in image (a) by using a piercing unit. c) Representative HR-TEM images of particle dimers and trimers illustrating the small interparticle gaps and cavities generated by this fabrication method.

For the characterization of the interparticle distances, which is a critical parameter for the efficient optical coupling of plasmon modes, an extensive collection of dimers and trimers was investigated by HR-TEM. Figure 1 c shows representative gaps between two particles, with values around 2 nm. Also, an average side length of 14–15 nm was measured for the triangular cavity formed by the assembly of three particles within trimers; this length is in good agreement with the geometrical prediction for 50 nm particles with an interparticle separation of 2 nm. Interestingly, these interparticle distances can be tuned either by decreasing the amount of PF68 (which leads to a decrease in the interparticle gap) or by decreasing the size of the nanoparticles (which decreases the cavity size). However, if the final aim of the cluster is optical sensing, the size of both gaps and particles should be engineered to maximize the optical enhancement while allowing the analyte to diffuse inside the cluster. With such considerations in mind, clusters with 2 nm gaps combine high optical coupling, generated by the strong electromagnetic fields from 50 nm particles,^23^ and sufficient space for diffusion of small- and medium-size analytes within the gaps.

The size of the particles is also appropriate to carry out optical and SERS characterization at the single-cluster level. To this end, dilute solutions of each sample were spin-coated onto a marked glass window. The slide surfaces were imaged with SEM to localize isolated clusters with different coordination numbers. Then, the areas containing the clusters were studied by dark-field optical microspectroscopy^24^ and SERS after retention of benzenethiol from the gas phase (Figure 2). This experiment allowed us to record the scattering spectrum and the optical enhancement from the same clusters.

**Figure 2:**
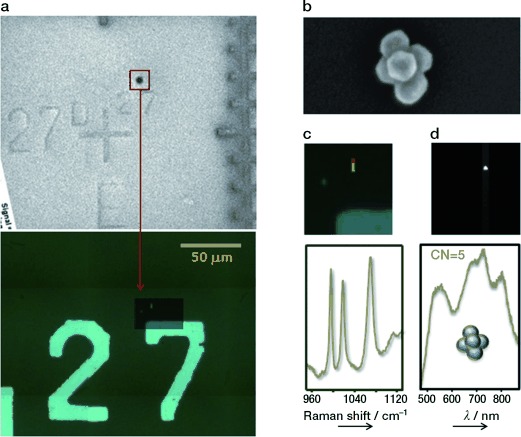
Schematic procedure for the optical and SERS characterization of single clusters. a) SEM and optical images of a marked glass surface spin-coated with one of the cluster samples. b) The cluster is localized and imaged with SEM. c, d) The region localized with SEM is imaged with Raman and dark-field spectroscopies.

Figure 3 shows measured and calculated^25^ (gold spheres of 50 nm in diameter were considered, with the metal described through its measured dielectric function^26^ and including a 0.6 nm coating of refractive index 1.3 to effectively represent the linking copolymer layer) Vis-NIR spectra of clusters with CNs ranging from 1 to 7. The agreement for low CNs is reasonably good, thus corroborating the optical behavior expected from plasmon hybridization and interaction between different gaps in the corresponding particle arrangements (see below). However, the comparison for CNs above 4 is less satisfactory, presumably owing to structural deformations of the clusters in the immersion oil (required to match the dielectric constants between the objective and the sample), thereby leading to more elongated arrangements that result in a strong redshift of the spectral weight. The spectra evolve towards more complex structures as the CN increases. Isolated nanospheres are dominated by a single dipolar-plasmon band at 543 nm, while the optical response of dimers is reminiscent of elongated anisotropic particles, characterized by two bands attributed to transversal and longitudinal plasmonic contributions. The latter is pushed towards the infrared region as a result of strong interparticle interaction through the gap, whereas the former is due to non-interacting transverse dipole plasmons that remain unaffected at the constituent individual particles. Trimers and higher-CN clusters exhibit more complex spectra, in which the short-wavelength transverse-dipole plasmon and the long-wavelength gap-mediated plasmon are modulated by the presence of additional spheres and the interaction among different gaps. While the dimer maintains a strong polarization dependence of its optical spectrum, a fully symmetrical trimer (*D*_3*h*_) has, by symmetry, a polarization-isotropic extinction spectrum. This results in the two experimentally observed bands, with a resolvable distortion revealed by a shoulder in the experiment; we attribute this distortion to symmetry breaking from slight differences in the shape of the gold particles. Theoretical predictions for the tetrahedral clusters (*T*_d_) show two main contributions, the electric-dipole resonance at 580 nm and the magnetic-dipole resonance around 800 nm.^27^ Analogously to the trimer, both resonances are distorted by symmetry breaking in the experimental spectra. Higher coordination numbers (5 (*D*_3*h*_ symmetry), 6 (*O_h_*), and 7 (*D*_5*h*_)) present complex calculated spectra that reflect an intricate interplay between electric and magnetic multipoles in the different spherical particles, conformed by the symmetry of each cluster configuration, although the experimental spectra are strongly affected by the immersion oil, as noted above.

**Figure 3:**
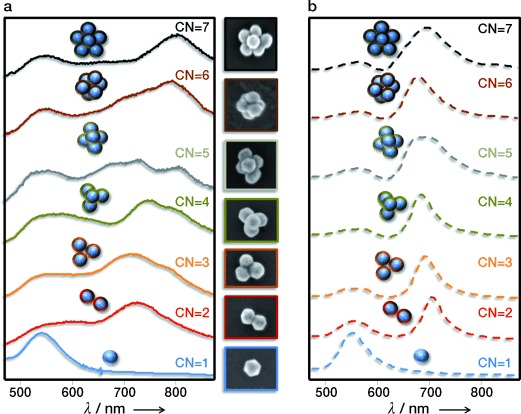
Optical response of nanoparticle clusters with coordination numbers from 1 to 7. a) Dark-field single-particle optical spectroscopy of the clusters. Next to it, SEM pictures of the corresponding clusters are shown. b) Electromagnetic simulations of the same clusters.

While much work has been invested into the optimization of dimer geometries to produce large electric field enhancements, as well as the resulting giant enhancement of SERS signals,^15^, 17c, 28 more complex geometries have not been thoroughly analyzed. As we show below, our results demonstrate that such higher coordination clusters can be used as efficient SERS platforms. To that end, we allowed adsorption of benzenethiol, a well-known SERS probe, from the gas phase^3^ onto the isolated clusters, previously localized on the marked slide. High-resolution confocal SERS was then used to study the clusters at the same positions where the dark-field microspectroscopy images had been acquired. Results for the measured optical enhancement of each structure are shown in Figure 4, normalized to the signal observed for a number of individual particles equal to the CN (enhancement=1). All clusters exhibit the strong SERS signals characteristic of benzenethiol (C-H bending at 1022 cm^−1^ and ring breathings at 1073 and 999 cm^−1^). Notably, individual single particles yielded no signal, and therefore their SERS spectrum was obtained from samples containing 27 well-separated particles within the sampled area. As expected, the SERS signals of a dimer and a trimer show an intensity increase around 100-and 300-fold, respectively. These enhancement factors (EFs) with respect to non-interacting individual particles, are well-understood and ascribed to the generation of optical hotspots within the gaps formed between the particles.2b, 29 For tetrahedral clusters, the SERS enhancement dramatically increases up to the level of three orders of magnitude. A similar abrupt step is observed for the trigonal bipyramid and the octahedra, while the pentagonal bipyramid reaches EFs close to 5000-fold. It should be stressed that the long-wavelength plasmon features observed in Figure 3 are displaced to the blue when the clusters are placed in air (see Figure S1 in the Supporting Information), so that the current illumination conditions (at 633 and 785 nm) are off-resonant. Furthermore, the near-field distributions show large field enhancement at the gaps between the particles (Figure 4 b). This finding is consistent with a tight-binding description of the optical response of these structures (see the Supporting Information), thus showing that the enhancement is associated with the gaps, and thus, other regions in the clusters are rather inactive for SERS. Therefore, as a first approximation, we interpret our results as receiving a contribution to the EF proportional to the number of gaps *G* in each cluster:


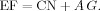
(1)

**Figure 4:**
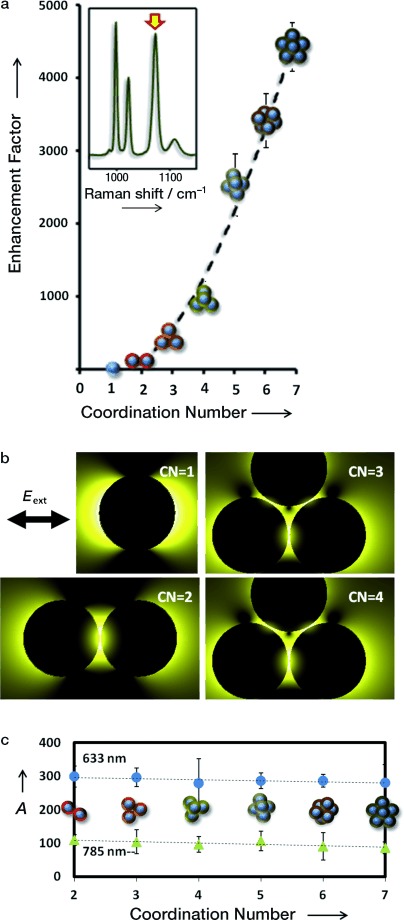
Optical enhancement of nanoparticle clusters with coordination numbers from 1 to 7. a) Comparison between the enhancement factors obtained for each sample, normalized to the enhancement produced by a single particle excited with a 633 nm laser line. Inset: SERS spectra of benzenethiol on the pentagonal bipyramid (CN 7). b) Near-field intensity (log thermal scale) for low-CN clusters in air at 633 nm along planes passing by the centers of the lower spheres. The maximum intensity relative to the incident intensity |*E*_ext_|^2^ is 14, 10 100, 5400, and 4000 for CN=1–4, respectively. c) Experimental data fitting with the following expression EF=CN + *A* *G*, where *G* represents the number of gaps and *A* is a constant factor reflecting the effective enhancement of the gap relative to the surface of an isolated sphere.

Notice that *G* increases strongly with the CN (e.g., *G*=1, 3, 6, 9, 12, and 15 for CN=2–7, respectively). The constant prefactor *A* equals the EF of the dimer relative to the individual particle. We show in Figure 4 c a fit of our data to this simple expression, which yields *A*=288.1±8.83 under 633 nm excitation and *A*=98.6±9.46 under 785 nm excitation. The smaller enhancement in the latter is expected, because the light wavelength is more displaced towards the red with respect to the airborne cluster plasmon bands (see the Supporting Information). Incidentally, the SERS EFs expected for these energies from the surface-integrated intensity enhancement calculated from MESME^30^ at the excitation and emitted wavelengths are 2092 and 297, respectively. The smaller values observed in the experiment can be attributed to a combination of nonlocal effects^31^ and nonuniform coverage of the gold surface by the analyte, so that the latter is not always present at the gap center, where the field enhancement is maximum.

In summary, we have shown that by using PF68 coating and emulsion clustering it is possible to produce plasmonic nanoparticle molecules with high symmetry and coordination index, and that they can be separated by applying density gradient centrifugation. PF68 produces narrow interparticle gaps with subsequent strong optical interactions while allowing the analytes to diffuse inside the gaps, where gigantic electric fields are generated, as we have shown by directly measuring the SERS enhancement in the clusters. Our geometrical nanostructures not only open a new path for the investigation of optical interactions between nanoparticles, but they also have great potential for applications to sensing and nonlinear nanophotonics.

## Supplementary material

Detailed facts of importance to specialist readers are published as ”Supporting Information”. Such documents are peer-reviewed, but not copy-edited or typeset. They are made available as submitted by the authors.
