# Supplementary material for: Organized Plasmonic Clusters with High Coordination Number and Extraordinary Enhancement in Surface-Enhanced Raman Scattering (SERS)
Source: Angew Chem Int Ed Engl. 2012 Nov 4;51(51):12688–93. doi: 10.1002/anie.201207019 (PMC3556689; doi:10.1002/anie.201207019)
Supplement: Supplementary file 1 [file anie0051-12688-SD1.pdf]

Angewandte Chemie
Eine Zeitschrift der Gesellschaft Deutscher Chemiker

Supporting Information

© Wiley-VCH 2012

69451 Weinheim, Germany

Organized Plasmonic Clusters with High Coordination Number and Extraordinary Enhancement in Surface-Enhanced Raman Scattering (SERS)**

Nicolas Pazos-Perez, Claudia Simone Wagner, Jose M. Romo-Herrera, Luis M. Liz-Marzán, F Javier García de Abajo, Alexander Wittemann,* Andreas Fery,* and Ramón A. Alvarez-Puebla**

anie_201207019_sm_miscellaneous_information.pdf

Supplementary Information:

Organized plasmonic clusters with high coordination number and extraordinary SERS enhancement

Nicolas Pazos-Pérez,[1,T] Claudia Simone Wagner,[2,T] Jose M. Romo-Herrera,[3,4] Luis M. Liz-Marzán,[4,5,6] F. Javier García de abajo,[7] Alexander Wittemann,[2,]* Andreas Fery,[1,]* Ramón A. Alvarez-Puebla[8,9]*

[1]Physikalische Chemie II, Universität Bayreuth, Universitätsstr. 30, 95440 Bayreuth, Germany; [2]Colloid chemistry, University of Konstanz, Universitaetsstrasse 10, 78464 Konstanz, Germany; [3] Centro de Nanociencias y Nanotecnología, UNAM, km 107 Carretera Tijuana-Ensenada, C.P. 22860, Ensenada B.C., México; [4]Departamento de Química-Física, Universidade de Vigo, 36310 Vigo, Spain; [5]Basque Foundation for Science, 48011 Bilbao, Spain. [6]Centre for Cooperative Research in Biomaterials (CIC biomaGUNE), Paseo de Miramón 182, 20009 San Sebastián, Spain; [7]Instituto de Quimica-Fisica Rocasolano-CSIC, Serrano 119, 28006, Madrid, Spain; [8]ICREA, Passeig Lluís Companys 23, 08010 Barcelona, Spain. [9]Department of Electronic Engineering & Center for Chemical Technology of Catalonia. Universitat Rovira i Virgili, Avda. Països Catalans, 2643007 Tarragona, Spain

*e-mail: alexander.wittemann@uni-konstanz.de; andreas.fery@uni-bayreuth.de; ramon.alvarez@urv.cat

[T]N.P.-P. and C.S.W. have contributed equally to the present work.

Experimental Methods

Chemicals. Trisodium citrate, tetrachloroauric(III) acid, sodium borohydride, ascorbic acid, and cetyltrimethylammonium bromide (CTAB), glycerol (all from Merk) toluene and cyclohexane (Riedel-de Haën), Pluronic®F-68 (PF68, Sigma) and benzenethiol (BT, Aldrich) were of analytical grade and used as received. Deionized water (Millipore Academic A10) was used throughout the experiments.

Synthesis of gold nanoparticles. The gold nanoparticles (50 nm) were synthesized by a seed-mediated approach, encompassing first the production of small gold seeds by preparing a solution (20 mL) containing trisodium citrate (2.5 10^{-4} M) and $HAuCl_4$ (2.5 10^{-4} M). The solution was vigorously stirred while sodium borohydride (0.1 M, 600 µL) was added. After stirring for 1 h, 500 µL of the seeds were added to a growth solution (250 mL) containing CTAB (0.1 M), $HAuCl_4$ (1.08 M, 1213 µL) and ascorbic acid (0.1 M, 1837 µL).

Stabilization and concentration of Au-CTAB with pluronic. To ensure particle-size monodispersity and stabilization in PF68, 250 mL of the Au solution (2.5$10^{-4}$ M) were centrifuged (8000 rpm, 27 C, 10 min) and redispersed three times. After each centrifugation, precipitate was dispersed in PF68 (1 w%) and supernatant discarded. The nanoparticle dispersion was left undisturbed during ~12 h at 27 C. Then, the precipitate was discarded and the supernatant centrifuged three more times (8000rpm, 27 C, 10 min), dispersing the precipitates in PF68 and discarding the supernatants. The final volume was 27 mL (1.25$10^{-3}$ M in gold)

Cluster fabrication from oil-in-water emulsions. The assembly of the gold nanospheres into clusters was accomplished through the agglomeration of the gold spheres while adsorbed onto toluene droplets. Briefly, suspensions of gold particles in 0.5 wt.% aqueous solution of PF68 were prepared. Subsequently, 0.4 mL toluene was added to 27 mL of these suspensions. Emulsification was accomplished with an ultrasonic horn that was dipped into the sample, so that the end of the sonotrode was touching the oil–water interphase. This procedure, in combination with the used geometry of the rosette cell (Bandelin RZ 3), ensured efficient mixing of the two phases immediately after sonication. The ultrasonication steps

were performed under ice-cooling using a high-shear homogenizer (Bandelin Sonoplus HD 3200, 200 W, probe KE 76). Three sonication steps, each for 5 min with 2 min rest in between, were performed at 30% amplitude and a frequency of 20 kHz. Packing of the particles into clusters was induced by evaporation of the toluene using a rotary evaporator (Heidolph Laborota 4000 efficient).

Cluster separation. Fractionation of the suspensions into clusters of the same coordination number was accomplished through density gradient centrifugation. Concentration gradients were built from 40 wt.% and 60 wt.% aqueous glycerol solutions using a gradient maker. The clusters of different sizes were separated by their sedimentation velocity during centrifugation at 5000 rpm for 6 min (Kontron Instruments Centrikon T-1080, Sorvall Surespin 630 rotor). The cluster fractions were isolated through a piercing unit (Kontron Instruments). Glycerol was removed by centrifugation and redispersion of the particles in deionized water.

Characterization. Vis-NIR spectra were recorded using an Agilent 8453 diode array spectrophotometer. Zeta potential was determined using a Malvern Instruments Zetasizer 2000. Scanning electron microscopy (SEM) images were obtained with a JEOL JSM 6700F field-emission microscope. High-resolution transmission electron microscopy (TEM) was conducted with a 2010 FEG-TEM microscope operating at an acceleration voltage of 200 kV. For single-particle optical characterization, diluted dispersions of clusters were spin-coated onto Lovins Field Finder Microslides (cat. 72266-01, Electron Microscopy Sciences) and localized and imaged with the SEM. Dark-field imaging and spectroscopy of the Au clusters was carried out on an inverted optical microscope (Nikon Eclipse TE-2000) equipped with an Acton SpectraPro 2150i monochromator and a Princeton Instruments Pixis 1024 charge coupled device (CCD). Samples were illuminated by white light from a 100 W tungsten lamp through a dark-field condenser (NA = 0.8). The scattered light was collected with a 40× objective (LD Plan-NEOFLUAR, NA = 0.6) and reflected to the entrance slit of the monochromator for imaging and spectroscopy. Scattering spectra from individual Au clusters were corrected by subtracting background spectra taken from the adjacent regions containing no Au nanoparticles. SERS experiments were conducted in a micro-Renishaw InVia Reflex system. The spectrograph uses a high resolution grating

(1200 grooves cm^{-1} for the NIR) with additional band-pass filter optics, a confocal microscope, a 2D-CCD camera and an automatized stage of 100 nm spatial resolution. Excitation was carried out by separately using laser lines at 632 nm and 785 nm. Benzenethiol was adsorbed in gas phase over the whole surface of the films by casting a drop of BT (0.1 M in ethanol) in a Petri box where the film was also contained. Surfaces were then mapped using the Renishaw StreamLine accessory with a step size of 1.2 μm in the same areas where SEM and dark-field data were collected. Acquisition times were set to 360 ms with power at the sample of 0.2 mW.

Theoretical calculations. Electromagnetic simulations were carried out using a multiple elastic-scattering of multipolar expansions (MESME) method,[30] in which the fields are expressed in terms of multipoles centered around each of the particles, and multiple scattering is carried out between them following a highly convergent Lanczos iterative procedure. The far-field amplitude was computed from the self-consistent expansion coefficients to generate the extinction cross-sections of Fig. 3, whereas the near fields were obtained by direct evaluation of the multipole expansion sums. Gold spheres of 50 nm in diameter were considered, with the metal described through its measured dielectric function[26] and including a 0.6 nm coating of refractive index 1.3 to effectively represent the linking copolymer layer. The gold-to-gold gap distance was set to 1.2 nm in all cases. The clusters were placed in a medium of refractive index 1.47 in Fig. 3, and in air in Fig. 4.

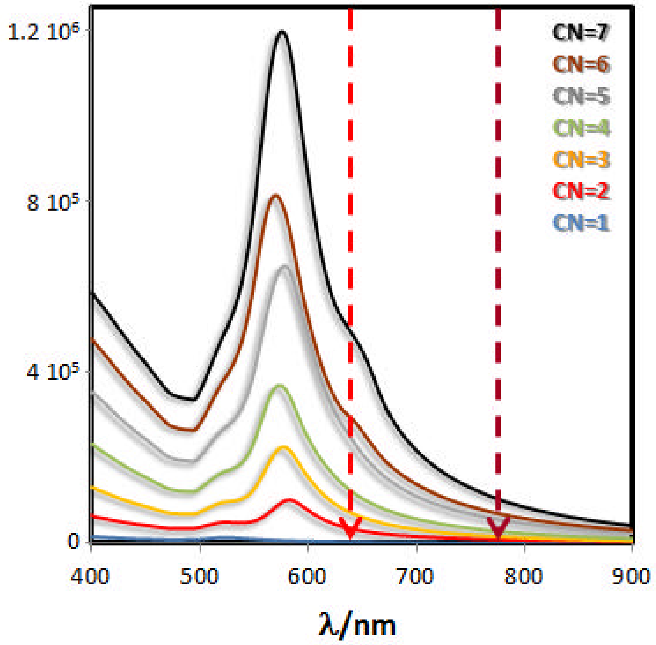

Figure S1. Spectra for clusters in air: Same as Fig. 3b of the main paper for particles placed in air. The plasmon resonances are blueshifted away from the wavelengths used for SERS in this work (dotted arrows).

Additional Theoretical Discussion

A tight-binding analysis of the plasmon resonances in the clusters produces revealing results to understand the long-wavelength features in the extinction spectra:

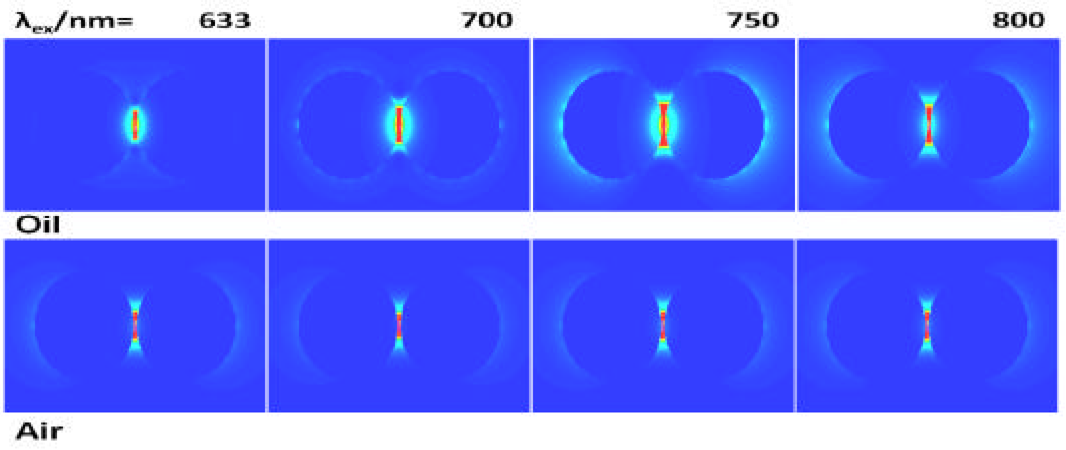

Figure S2. Near-field maps for a CN=2 cluster in oil and air upon excitation with ligth from 633 to 800 nm. The plots show ecuatorial cuts with the external field along the gap.

We use the +− dipole mode confined to the gap of the dimer in oil as the unit *wave function* of our tight-binding analysis, at wavelength λ~720 nm (energy E_0~1.7 eV).

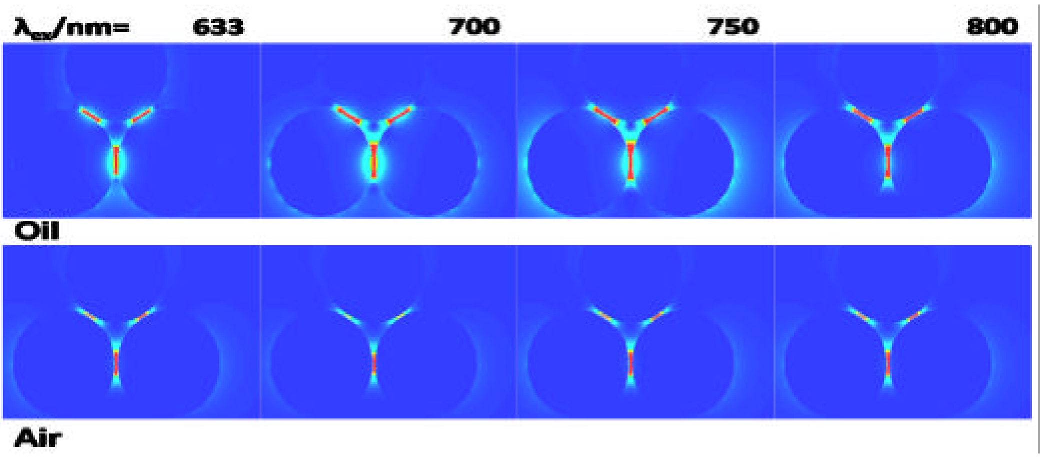

Figure S3. Near-field maps for a CN=3 cluster in oil and air upon excitation with ligth from 633 to 800 nm. The plots show ecuatorial cuts of the three spheres.

The trimer consists of three gaps, with an inter-gap interaction Δ. This leads to modes of energies determined by the diagonalization of the interaction matrix, consisting of zeros in the diagonal and with all off-diagonal elements set to Δ. This produces a symmetric mode with energy $E_0+2\Delta$ and equal weights in all gaps, as well as two degenerate modes of energy $E_0-\Delta$. The symmetric mode does not have a net dipole moment, and thus, it should not couple efficiently to the external light. It is actually absent from the calculations shown in Fig. 3b of the main paper. In contrast, the degenerate modes have a dipole moment and are responsible for the prominent long-wavelength feature, which is blueshifted in the trimer with respect to the dimer, thus suggesting an attractive interaction between gaps ($\Delta<0$), consistent with the +– gap-mode charge structure. The symmetric mode shows up in the spectra at its expected position (redshifted with respect to the dimer by twice as much as the blueshift of the bright modes), presumably as a result of asymmetries in the actual cluster.

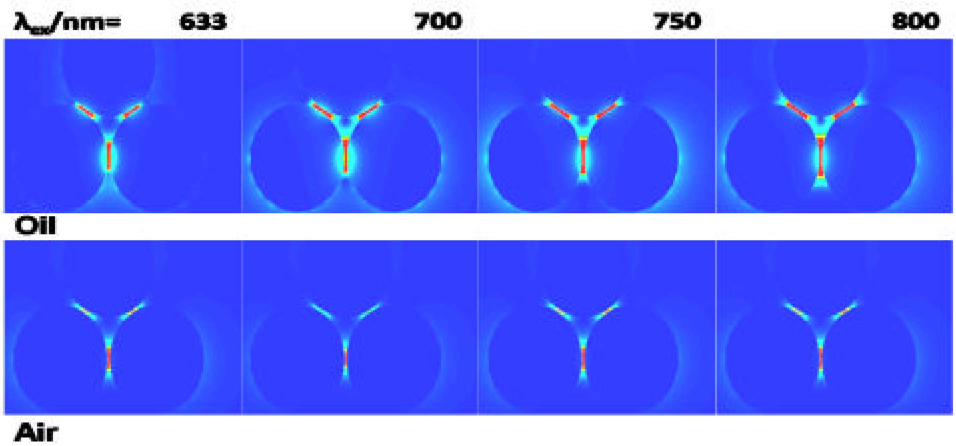

Figure S4. Near-field maps for a CN=4 cluster in oil and air upon excitation with ligth from 633 to 800 nm. The plots show ecuatorial cuts of the three lower spheres.

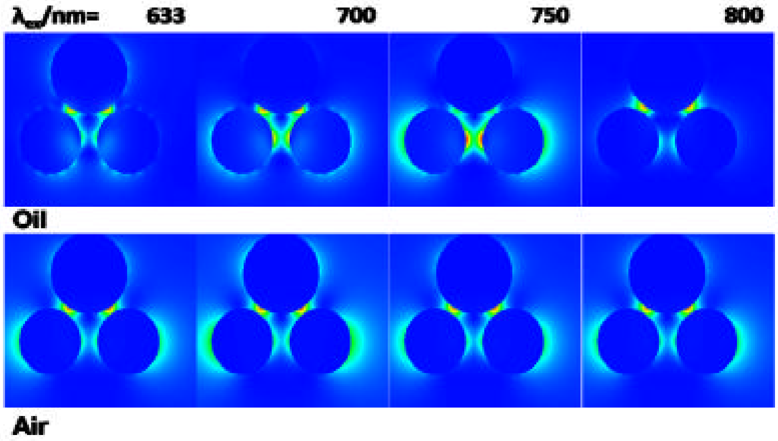

Figure S5. Near field maps for a CN=4 cluster in oil and air upon excitation with ligth from 633 to 800 nm. Vertical cuts.

From a similar analysis of the CN=4 cluster, we find two sets of three degenerate modes at energies E_0–2Δ and E_0+2Δ (six modes in total, as we have six gaps in tetrahedral cluster). The former are the only ones that display a net dipole, and thus, they produce a dominant spectral feature, which is correctly redshifted with respect to the dimer by twice as much as the trimer plasmon in the calculated spectrum of Fig. 3b of the main paper. The experimental spectrum for the tetrahedron (Fig. 3a of main paper) is already showing some signatures of distortion produced by the immersion oil.

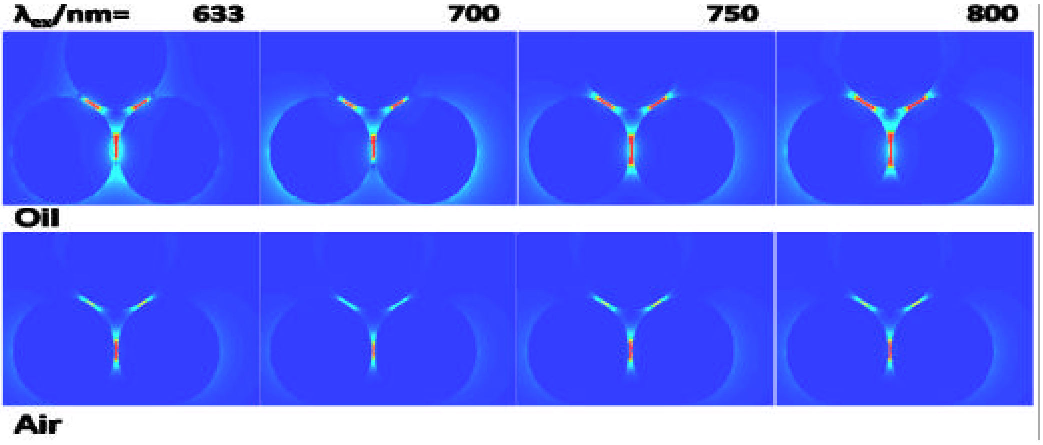

Figure S6. Near field maps for a CN=5 cluster in oil and air upon excitation with ligth from 633 to 800 nm. Ecuatorial cuts of the three central spheres.

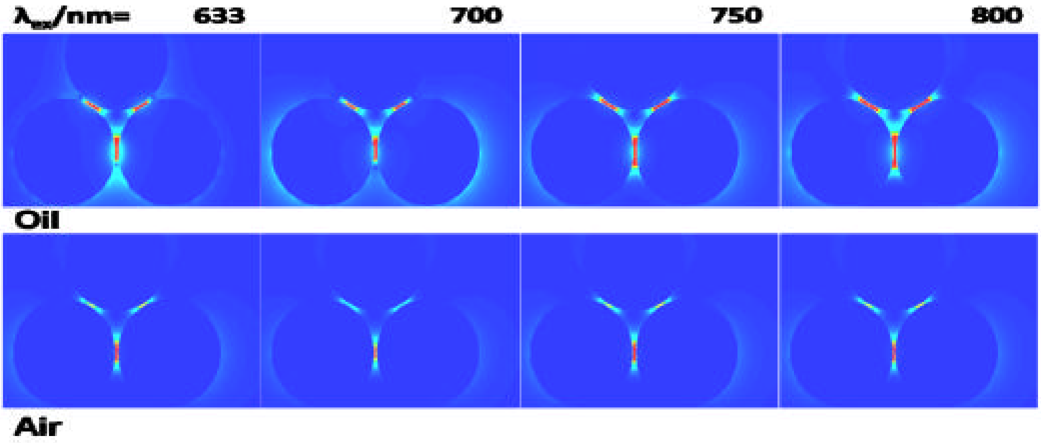

Figure S7. Near field maps for a CN=5 cluster in oil and air upon excitation with ligth from 633 to 800 nm. Vertical cuts.

A similar analysis can be easily performed for higher CN clusters. This analysis, and the agreement with experiment for low CN, indicates that the gap modes form a good basis set to describe the optical behavior of the clusters. Higher CN clusters seem to be deformed by the immersion oil, and thus direct comparison with experiment is not possible, but the tight-binding model produces very good results compared with full MESME calculations. This study also demonstrates that the gaps act rather independently under off-resonance illumination (i.e., for the conditions used to acquire ours SERS spectra with the clusters in air), and thus, the number of gaps is the main magnitude that determines the SERS enhancement factor of a given cluster.
